# Molecular mechanism of EAG1 channel inhibition by imipramine binding to the PAS domain

**DOI:** 10.1016/j.jbc.2023.105391

**Published:** 2023-10-28

**Authors:** Ze-Jun Wang, Mahdi Ghorbani, Xi Chen, Purushottam B. Tiwari, Jeffery B. Klauda, Tinatin I. Brelidze

**Affiliations:** 1 Department of Pharmacology and Physiology, Georgetown University Medical Center, Washington, District of Columbia, USA; 2 Department of Chemical and Biomolecular Engineering, University of Maryland, College Park, Maryland, USA; 3 Department of Oncology, Georgetown University Medical Center, Washington, District of Columbia, USA; 4 Institute for Physical Science and Technology and Biophysics Graduate Program, University of Maryland, College Park, Maryland, USA

**Keywords:** KCNH1, EAG, Kv10.1 channels, potassium channel, inhibitor, inhibition mechanism, ligand-binding protein, gating

## Abstract

Ether-a-go-go (EAG) channels are key regulators of neuronal excitability and tumorigenesis. EAG channels contain an N-terminal Per-Arnt-Sim (PAS) domain that can regulate currents from EAG channels by binding small molecules. The molecular mechanism of this regulation is not clear. Using surface plasmon resonance and electrophysiology we show that a small molecule ligand imipramine can bind to the PAS domain of EAG1 channels and inhibit EAG1 currents *via* this binding. We further used a combination of molecular dynamics (MD) simulations, electrophysiology, and mutagenesis to investigate the molecular mechanism of EAG1 current inhibition by imipramine binding to the PAS domain. We found that Tyr71, located at the entrance to the PAS domain cavity, serves as a “gatekeeper” limiting access of imipramine to the cavity. MD simulations indicate that the hydrophobic electrostatic profile of the cavity facilitates imipramine binding and *in silico* mutations of hydrophobic cavity-lining residues to negatively charged glutamates decreased imipramine binding. Probing the PAS domain cavity-lining residues with site-directed mutagenesis, guided by MD simulations, identified D39 and R84 as residues essential for the EAG1 channel inhibition by imipramine binding to the PAS domain. Taken together, our study identified specific residues in the PAS domain that could increase or decrease EAG1 current inhibition by imipramine binding to the PAS domain. These findings should further the understanding of molecular mechanisms of EAG1 channel regulation by ligands and facilitate the development of therapeutic agents targeting these channels.

Ether-a-go-go (EAG) channels, also known as Kv10.1 and KCNH1 channels, contribute to the regulation of neuronal excitability (1, 2, 3) and gain-of-function genetic mutations in EAG channels are associated with severe neurological disorders (4, 5, 6, 7, 8). In addition to their function in the brain, EAG channels are upregulated in cancer (9, 10, 11, 12, 13) and inhibition of their activity has been shown to reduce cancer cell proliferation (14, 15, 16).

EAG channels belong to the KCNH family of voltage-gated potassium channels that also include EAG-related gene (ERG) and EAG-like K+ (ELK) subfamilies (17, 18). Similar to other voltage-gated potassium channels, EAG channels are assembled from four subunits, each containing six membrane-spanning segments (S1–S6) (17, 19, 20). The S1–S4 segments form a voltage-sensor domain (VSD) and segments S5–S6 form a pore-domain (PD) (Fig. 1*A*). The centrally located pore of the channel is formed from the PDs of all four subunits, with the p-loops between the S5 and S6 segments lining the selectivity filter of the channel. Each subunit of EAG channels also contains an N-terminal Per-Arnt-Sim (PAS) domain and a C-terminal cyclic nucleotide-binding homology (CNBH) domain (17, 20, 21). The PAS and CNBH domains from adjacent subunits form inter-subunit interactions in EAG channels (20, 22, 23).

**Figure 1:**
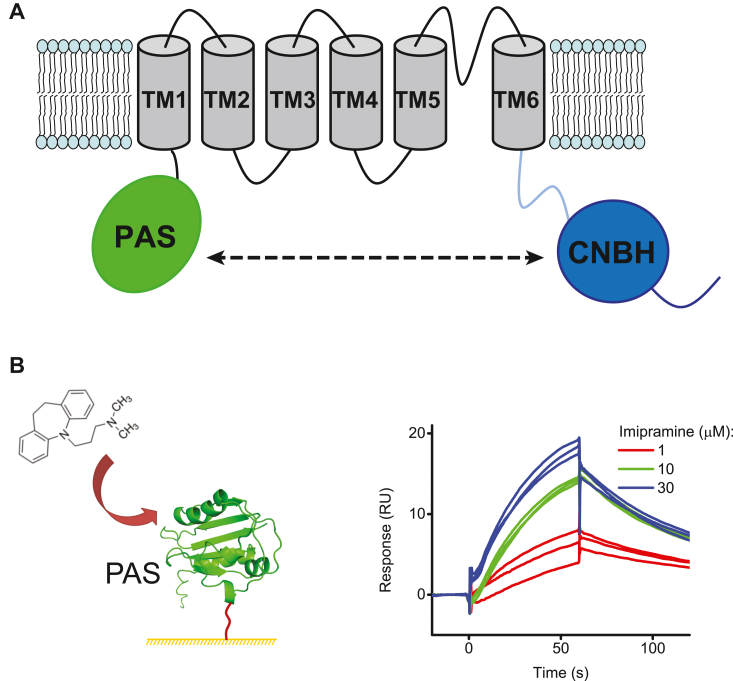
**Imipramine binds to the PAS domain of EAG1 channels.***A*, topology of an EAG1 channel monomeric subunit. The PAS domain is shown in *green*, membrane-spanning segments in *gray*, C-linker in *cyan*, and CNBH domain in *blue*. The *dashed two-sided arrow* signifies interactions between the PAS and the CNBH domains from adjacent subunits in an EAG1 channel tetramer. *B*, schematic of the PAS domain immobilized on the CM5 chip using amine coupling and SPR sensorgrams recorded for the immobilized PAS domain in response to the application of imipramine at 1, 10, and 30 μM concentrations.

In addition to forming the intersubunit interactions, it has been shown that PAS domains of EAG channels bind small molecule ligands chlorpromazine and undecylenic acid (24, 25), and PAS domains of related ERG3 channels bind heme (26). While it has been proposed that these small molecule ligands cause inhibition of currents through these channels *via* the binding to the PAS domains, so far, the only evidence in support of this mechanism is that the deletion of the PAS domain decreases the inhibition of EAG currents by chlorpromazine (25). However, deletion of an entire domain could have unforeseen effects on the channel gating that could mask the mechanism of the small molecules effect on EAG and related channels.

Here we identified, for the first time, specific residues on the PAS domain of EAG1 channels that facilitate or limit EAG current inhibition by small molecule ligands. We used surface plasmon resonance (SPR) to show that a small molecule ligand imipramine directly binds to the PAS domain of EAG1 channels. We then used electrophysiology and site-directed mutagenesis, guided by molecular dynamics (MD) simulations, to further examine the molecular mechanism of EAG1 current inhibition by imipramine.

We found that deletion of the PAS domain substantially decreased EAG1 current inhibition by imipramine, suggesting that the effect of imipramine is, at least in part, mediated *via* binding to the PAS domain. Docking of imipramine into the PAS domain cavity and accompanied MD simulations identified Tyr71, located at the entrance to the cavity, as a “gatekeeper” residue, limiting access of imipramine to the binding site inside the cavity. Consistent with this, substitution of Tyr71 with glycine and valine increased EAG1 current inhibition by imipramine, while substitution with phenylalanine had no effect on the current inhibition by imipramine. MD simulations indicated that the hydrophobic profile of the cavity facilitates the binding of imipramine and *in silico* mutations of four PAS domain lining hydrophobic residues to negatively charged glutamates decreased imipramine binding. Computational modeling-driven mutagenesis studies of residues lining the PAS domain cavity identified residues D39 and R84 as essential for the EAG1 current inhibition by imipramine binding to the PAS domain. Consistent with this, substitutions of D39 and R84 residues with glycine substantially decreased EAG current inhibition by imipramine.

The residual inhibition of EAG1 channels lacking the PAS domain, combined with the previously reported inhibition of EAG1 channels by imipramine *via* an open-pore block (27), indicates that imipramine inhibits EAG1 currents by a dual mechanism: by binding to the PAS domain and by blocking the conduction pore. Taken together, our results shed light on the molecular mechanism of EAG1 current inhibition by the PAS domain small molecule binders.

## Results

### Imipramine binds to the PAS domain of EAG1 channels

To test if imipramine can directly bind to the PAS domain of EAG1 channels we immobilized purified isolated PAS domains of EAG1 channels on the CM5 sensor ship using amine coupling and determined the SPR response over the range of concentrations of imipramine injected over the immobilized PAS domains. The SPR response increased with the increase in the imipramine concentration (Fig. 1*B*). Because of non-specific binding of imipramine to the CM5 sensor chip at high concentrations, we were unable to obtain the complete concentration dependence of the binding necessary to determine the binding affinity. However, the concentration-dependent increase in the SPR response for the tested imipramine concentrations indicates that similar to chlorpromazine, imipramine directly binds to the PAS domain of EAG1 channels.

### Imipramine inhibits EAG1 currents and deletion of the PAS domain decreases the inhibition

**Figure 2:**
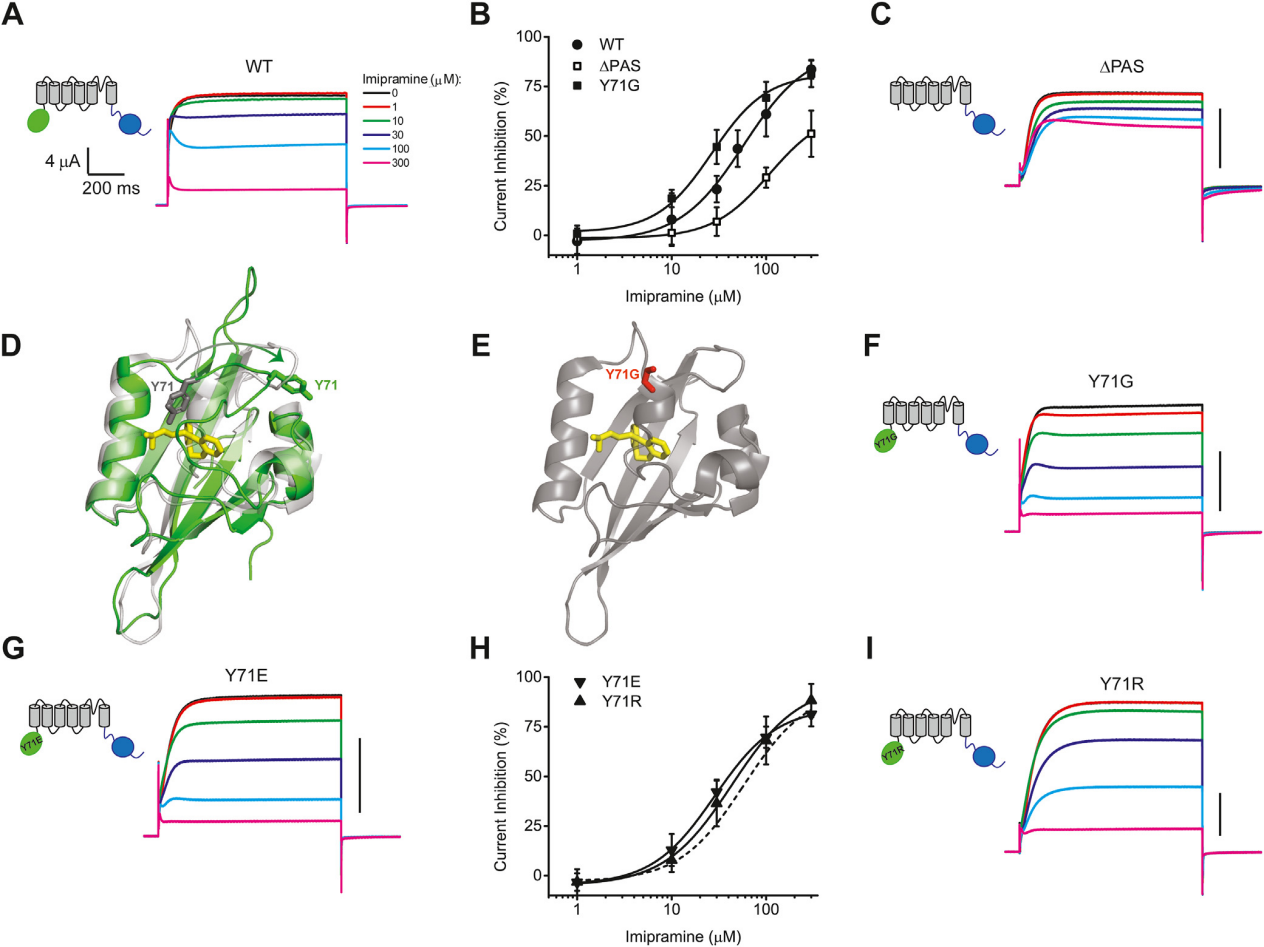
**Y71 restrictsbinding of imipramine to the PAS domain cavity.** Currents from WT (*A*), ΔPAS (*C*), Y71G (*F*), Y71E (*G*), and Y71R (*I*) mutant EAG1 channels were recorded at +50 mV with two-electrode voltage clamp in the presence of the indicated imipramine concentrations. *B*, plots of the averaged percentage of steady-state current inhibition *versus* imipramine concentration for WT (*filled circles*, n = 5), ΔPAS (*open squares*, n = 5) and Y71G (*filled squares*, n = 4) mutant EAG1 channels. *D*, imipramine bound inside the PAS domain cavity after REST simulations. The imipramine-bound PAS domain structure is shown in the *green ribbon* representation with the Y71 residue in *green sticks* and imipramine in *yellow*. The ligand-free structure of the PAS domain is shown in the *transparent grey color* with the Y71 residue in *sticks*. The *curved arrow* indicates a possible transition of Y71 from ligand-free to ligand-bound states in order to give access to imipramine to the PAS domain cavity. *E*, ligand-free PAS domain structure with glycine substituted for Y71 and imipramine docked in the same pose as in *D*. *H*, plots of the averaged percentage of steady-state current inhibition *versus* imipramine concentration for Y71E (*downward pointing triangles*, n = 5) and Y71R (*upward pointing triangles*, n = 4) mutant EAG1 channels. The *solid lines* in *B* and *H* represent fits of the data with the Hill equation. The *dashed line* in *H* corresponds to the fit with the Hill equation for WT channels from *B*. The IC_50_ values and the corresponding statistical analysis can be found in Table 1. The data in *B* and *H* are presented as mean ± SD. Scale bars in *C*, *F*, *G*, *I*: 4 μA.

To determine the functional effect of imipramine on EAG1 channels we recorded currents from EAG1 channels using the two-electrode voltage clamp (TEVC) technique in the absence and presence of imipramine. Imipramine inhibited EAG1 currents in a concentration-dependent manner with the IC_50_ of 58.1 ± 9.7 μM at +50 mV (Fig. 2, *A* and *B*, and Table 1). To further determine the contribution of imipramine binding to the PAS domain to the current inhibition, currents were recorded from mutant EAG1 channels lacking the N-terminal PAS domain (ΔPAS) in the absence and presence of imipramine. The deletion of the PAS domain substantially increased the IC_50_ of the current inhibition by imipramine and also decreased the efficacy (maximal inhibition) observed at the highest examined ligand concentrations, suggesting that direct binding of imipramine to the PAS domain is contributing to the EAG1 current inhibition (Fig. 2, *B* and *C*, and Table 1). The residual inhibition by imipramine of EAG1 channels lacking the PAS domain is most likely due to the secondary inhibitory mechanism *via* the pore block, as proposed before (27).

**Table 1:** IC_50_ (μM) for imipramine inhibition at +50 mV for WT and indicated mutant EAG1 channels

Channel	IC_50_ (μM)	*p* Value
WT	58.1 ± 9.7 (5)	
ΔPAS	108.6 ± 12.6 (5)^a^	0.0131
Y71G	26.7 ± 3.2 (4)^a^	0.0279
Y71V	31.3 ± 1.8 (6)^a^	0.0153
Y71F	58.0 ± 3.7 (6)	0.9920
Y71E	28.5 ± 1.4 (5)^a^	0.0166
Y71R	43.4 ± 7.5 (4)	0.2892
D39G	79.6 ± 5.4 (6)	0.0731
V80G	57.1 ± 3.1 (6)	0.9176
V83G	44.3 ± 2.6 (6)	0.1685
R84G	92.6 ± 2.7 (6)^a^	0.0047
F87G	42.8 ± 0.6 (5)	0.1541
F130G	52.8 ± 5.0 (6)	0.6211
D39G/R84G	142 ± 28.5 (6)^a^	0.0060

a *p* < 0.05 by Student’s *t* test. *p* < 0.05 was considered statistically significant. *p*-values represent significance of Student’s t tests used to compare the IC_50_ for imipramine inhibition at +50 mV for WT and indicated mutant channels. The number of averaged recordings from different oocytes is indicated in parentheses. The difference between IC50 for ΔPAS and R84G mutants was not statistically significant (*p* value of 0.2067). The difference between IC50 for ΔPAS and D39G/R84G mutants was not statistically significant (*p* value of 0.0718).

### Y71 restricts access of imipramine to the PAS cavity

To determine the structural basis of imipramine binding to the PAS domain, we performed computational modeling. While KCNH are the only known ion channels to include PAS domains as part of their amino acid sequence, PAS domain fold is quite common in non-ion channel proteins. In the PAS domains of other proteins, ligands typically bind inside the PAS domain cavity formed by the antiparallel β-strands sandwiched between the α-helices (28, 29, 30). However, the initial docking of imipramine into the PAS domain cavity using AutoDock Vina (31) indicated that Y71 blocks the entrance to the cavity for imipramine (shown in grey in Fig. 2, *D*). To sample thermodynamically available conformations, we used replica exchange solute tempering (REST2) (32) to elucidate the ligand-accessible PAS domain cavity. The REST2 simulations indicated that in the ligand-accessible conformation, Y71 swings away from the binding pocket, no longer blocking imipramine binding inside the cavity (shown in green in Fig. 2*D*). Consistent with these results, replacing the tyrosine at position 71 with a smaller glycine and valine increased EAG1 current inhibition for imipramine (Figs. 2, *B*, *E* and *F* and S1, *A* and *C*, and Table 1), while replacing the tyrosine with structurally similar phenylalanine had no statistically significant effect on imipramine inhibition (Fig. S1, *B* and *D*). These results strongly suggested that Y71 functions as a “gate-keeper” residue, restricting access of imipramine to the PAS domain cavity.

To further investigate if the charge at position 71 may have an effect on EAG1 current inhibition by imipramine, Y71 was substituted for either glutamate or arginine. Imipramine inhibition of EAG channels with Y71E or Y71R mutation was not statistically significantly different from each other, suggesting that the effect of Y71 on EAG1 current inhibition by imipramine does not involve electrostatic interactions.

### The hydrophobic profile of the PAS cavity facilitates imipramine binding

**Figure 3:**
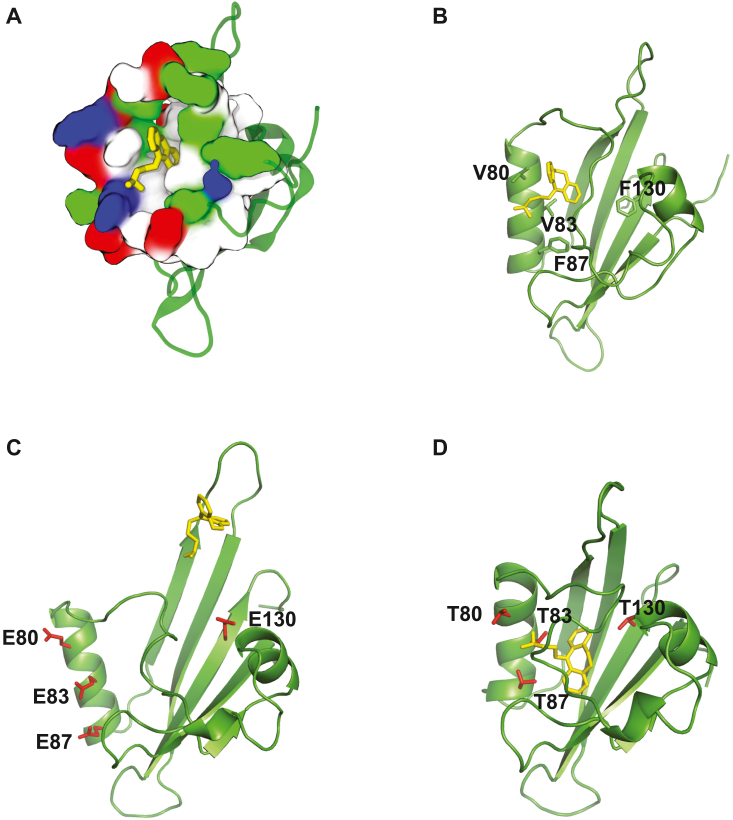
**Hydrophobic profile of the PAS domain cavity facilitates imipramine binding.***A*, electrostatic surface profile of the PAS domain cavity. Acidic residues are shown in *red*, basic residues in *blue*, hydrophobic residues in *white* and polar residues in *green*. *B*–*D*, a representative binding pose of imipramine (*yellow*) inside the WT (*B*), 4E (*C*) and 4T (*D*) mutant PAS domain cavity determined with 100 ns MD simulations.

The analysis of the electrostatic profile of the PAS domain indicated that the PAS domain cavity is lined with predominantly hydrophobic residues (Fig. 3*A*). The hydrophobic profile of the cavity should facilitate the binding of the hydrophobic tricyclic ring structure of imipramine. To investigate the dynamics of imipramine binding, the initial poses for imipramine binding obtained with the REST2 simulations were subjected to 100 ns MD simulations. The MD simulations indicated that imipramine remained in the binding pocket with its three rings facing the inside of the PAS domain cavity (Fig. 3*B*).

To further investigate the contribution of the hydrophobic profile of the cavity we *in silico* generated mutant PAS domains with the cavity lining hydrophobic residues V80, V83, F87 and F130 mutated to glutamates (4E mutant) or phenylalanines (4T mutant). The mutant PAS domain structures were generated using Amber force field with the initial structures taken from the REST2 simulations. Imipramine was docked into the mutant PAS domains and subjected to 100 ns MD simulations. For the 4E mutant PAS domain imipramine drifted away from the cavity after the first 10 ns (Fig. 3*C*), while for the 4T mutant PAS domain imipramine stayed inside the cavity for the duration of the simulation (Fig. 3*D*). We next calculated imipramine binding free energies for the wild-type (WT) and two mutant PAS domains using MM-PBSA (33). Different components of the binding free energy, including van der Waals (VDW), electrostatic, polar, and non-polar solvation energies, are given in Table 2. The breakdown of the binding free energy indicates that the binding is driven by hydrophobic interactions between the hydrophobic residues in the cavity and the hydrophobic rings of imipramine. Consistent with this, the imipramine binding free energy for the 4E mutant PAS domain was significantly reduced, while for the 4T mutant, it was the same as for the WT PAS domain (Table 2). Taken together, the results of the MD simulations for the WT and two mutant PAS domains suggest that the hydrophobic profile of the cavity facilitates imipramine binding.

**Table 2:** Imipramine binding free energies (in kcal/mol) calculated with MM-PBSA

PAS domain	VDW	Elec	Polar solv	Non-polar solv	Total
WT	−42.72 ± 0.67	−0.20 ± 0.1	12.59 ± 0.17	−4.41 ± 0.02	−34.74 ± 0.25
4E	−23.40 ± 0.31	−0.81 ± 0.07	8.25 ± 0.23	−2.78 ± 0.02	−18.74 ± 0.31
4T	−41.96 ± 0.30	−0.80 ± 0.04	11.96 ± 0.11	−4.18 ± 0.01	−34.98 ± 0.28

Abbreviations: elec, electrostatic; non-polar solv, non-polar solvation; polar solv, polar solvation; VDW, Van der Waals.

Total energies were computed with MM-PBSA.

### Residues D39 and R84 located in the PAS domain cavity are essential for EAG1 current inhibition by imipramine

**Figure 4:**
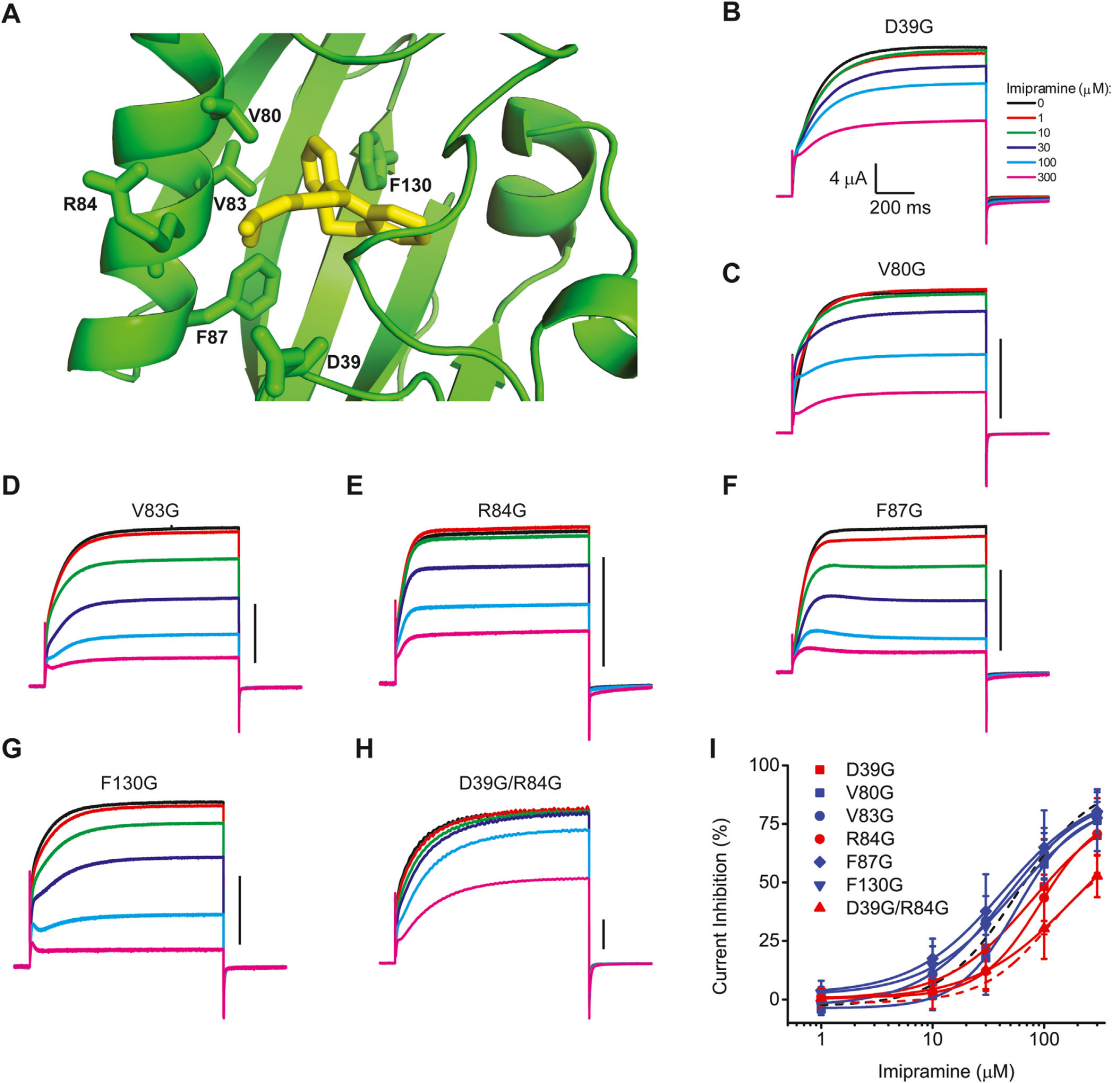
**Cavity-flanking residues D39G and R84G are essential for EAG1 current inhibition by imipramine binding to the PAS domain.***A*, close-up view of imipramine bound inside the PAS domain cavity with the residues poised to interact with the ligand shown in *stick* representations. Currents from D39G (*B*), V80G (*C*), V83G (*D*), R84G (*E*), F87G (*F*), F130G (*G*), and D39G/R84G (*H*) mutant EAG1 channels recorded at +50 mV with two-electrode voltage clamp in the presence of the indicated imipramine concentrations. Scale bars in (*C*–*H*): 4 μA. *I*, plots of the averaged percentage of steady-state current inhibition *versus* imipramine concentration for D39G (*red squares*), V80G (*blue squares*), V83G (*blue circles*), R84G *(red circles*), F87G (*blue diamonds*), F130G (*blue downward pointing triangles*) and D39G/R84G (*red upward pointing triangles*) mutant EAG1 channels recorded at +50 mV with two-electrode voltage clamp in the presence of the indicated imipramine concentrations. (n ≥ 5 for each condition). The *solid lines* represent fits of the data with the Hill equation. The *black dashed line* corresponds to the fit with the Hill equation for WT channels from Figure 2*B* and *red dashed line* to the fit for ΔPAS channels. The IC_50_ values and the corresponding statistical analysis can be found in Table 1. The data in *I* are presented as mean ± SD.

The computational modeling suggested that residues D39, V80, V83, R84, F87, and F130 in the PAS domain cavity may be contributing to the coordination of imipramine inside the PAS cavity (Fig. 4*A*). To investigate the contribution of these residues to the imipramine inhibition of EAG1 currents, they were substituted one by one for glycine, and currents from the mutant channels were recorded over the range of imipramine concentrations (Fig. 4, *B*–*H*). Substituting glycine for R84 showed a statistically significant increase in the IC_50_ of EAG1 current inhibition and this increase was statistically the same as the one observed for the ΔPAS mutation (Fig. 4*I* and Table 1). The other five mutations did not have a substantial effect on the IC_50_ of imipramine inhibition (Fig. 4*I* and Table 1). Interactions mediated by van der Waals forces and potential π−π stacking are energetically weaker than electrostatic interactions. Therefore, the absence of an effect on the IC_50_ for the individual valine and phenylalanine substitutions is not surprising. MD simulations suggest that these hydrophobic residues are collectively contributing to the ligand coordination and binding. To experimentally test this possibility, we introduced double mutations F87G/F130G, V80G/F87G, and F87G/F130G. Unfortunately, these double mutant channels did not generate any detectable currents. Therefore, we were unable to experimentally determine the collective contribution of the hydrophobic residues to the imipramine binding and coordination in the PAS domain cavity.

Although the D39G mutation did not change the IC_50_ in a statistically significant manner, it caused a similar decrease in the current inhibition as R84G mutation at high imipramine concentrations (Fig. 4*I*). To further examine the contribution of D39 and R84 residues to the current inhibition, we generated a double mutant D39G/R84G EAG1 channels. The double mutant channels generated currents and showed an even larger decrease in the current inhibition at 300 μM imipramine concentration than the one observed for the individual mutations (Fig. 4, *H* and *I*, and Table 1). Moreover, the decrease in the current inhibition was comparable to the one observed for ΔPAS mutant channels. Taken together, these results suggest that D39G and R84G residues are essential for EAG1 current inhibition by imipramine binding to the PAS domain.

**Figure 5:**
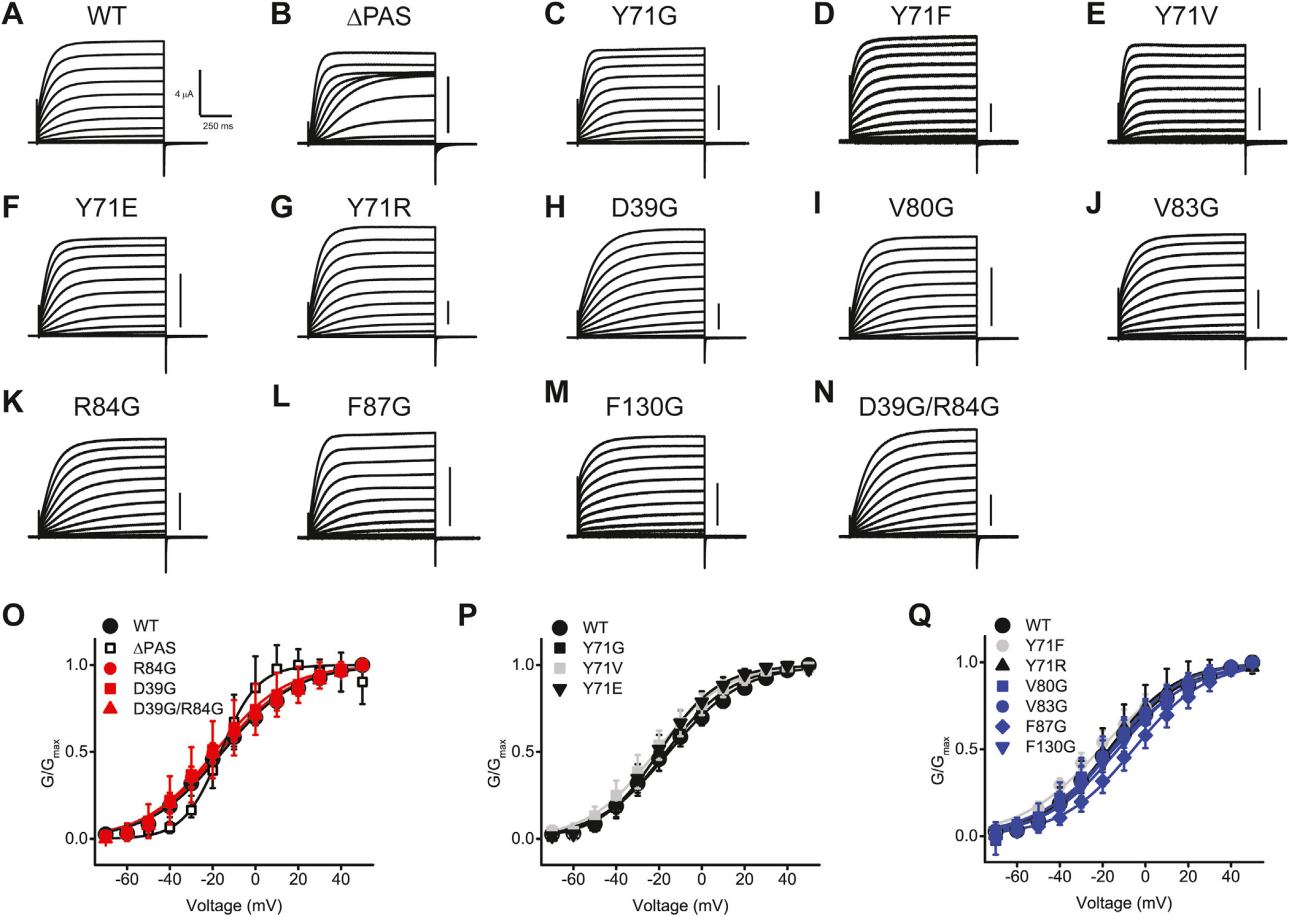
**Voltage dependence and the current profile of the WT and mutant EAG1 channels used in the study.***A*–*N*, representative current traces recorded with two-electrode voltage clamp. *O*–*Q*, plots of the averaged normalized conductance *versus* voltage for the WT and indicated mutant channels that increased the IC_50_ for imipramine inhibition of EAG1 currents (*O*), decreased IC_50_ (*P*) or had no effect on IC_50_ (*Q*). The size of the WT data points (*filled black circles*) in *O*–*Q* was increased relative to other symbols to prevent a complete overlap with the data points for the mutant channels. The averaged V_1/2_ values, n of experiments, and statistical analysis can be found in Table 3. The data in *O*–*Q* are presented as mean ± SD. Scale bars in (*B*–*N*): 4 μA.

It is possible that the considered mutations in EAG1 channels substantially alter channel gating obscuring the interpretation of the mechanisms of imipramine inhibition. Contrary to this possibility, the current profile in the absence of the inhibitors was very similar for all mutant channels tested, except ΔPAS mutant that had been shown to affect the voltage-dependence of EAG1 channels (34, 35) (Fig. 5, *A*–*N*). Most of the mutations that affected IC_50_ of EAG1 channel inhibition by imipramine had no statistically significant effect on the half-maximal activation voltage (V_1/2_) of the EAG1 channel, except for Y71E and D39G that generated a mild <5 mV shift to more depolarized potentials in V_1/2_ and Y71V that generated <5 mV shift to more hyperpolarized potentials (Fig. 5, *O*–*P*, and Table 3). Three mutations that did not affect EAG1 current inhibition, Y71F, F87G, and F130G, shifted V_1/2_ to more hyperpolarized potentials (Fig. 5*Q*). Importantly, Y71G that increased EAG1 current inhibition by imipramine and D39G/R84G double mutation that decreased EAG1 current inhibition had no effect on V_1/2_ (Fig. 5, *O* and *P*). These results indicate that the considered mutations specifically affected EAG1 channel inhibition by imipramine without causing changes in imipramine-independent channel gating mechanisms.

**Table 3:** V_1/2_ (mV) values for WT and mutant channels and the corresponding *p* values by Student’s *t* test

Channel	V_1/2_ (mV)	*p* Value
WT	−15.3 ± 0.7 (5)	
ΔPAS	−16.3 ± 0.8 (5)	0.3744
Y71G	−16.7 ± 0.7 (5)	0.1950
Y71F	−21.1 ± 1.0 (6)^a^	0.0014
Y71V	−21.4 ± 0.5 (6)^a^	0.0001
Y71E	−19.9 ± 0.5 (5)^a^	0.0007
Y71R	−17.1 ± 0.6 (5)	0.0867
D39G	−18.8 ± 0.7 (6)^a^	0.0067
V80G	−14.8 ± 1.1 (6)	0.7237
V83G	−15.6 ± 0.6 (6)	0.7508
R84G	−17.0 ± 0.9 (6)	0.1831
F87G	−5.6 ± 0.6 (5)^a^	0.0001
F130G	−12.7 ± 0.6 (6)^a^	0.0195
D39G/R84G	−16.8 ± 1.2 (6)	0.3340

a *p* < 0.05 by Student’s *t* test. *p* < 0.05 was considered statistically significant. *p*-values represent significance of Student’s t tests used to compare the V_1/2_ values for WT and indicated mutant channels. The number of averaged recordings from different oocytes is indicated in parentheses.

## Discussion

In this study, we showed that imipramine inhibits EAG1 channels through binding to their PAS domains and identified specific PAS domain residues that facilitate or limit EAG1 current inhibition by imipramine. MD simulations coupled with electrophysiology and site-directed mutagenesis indicated that the PAS domain residue Y71 functions as a “gate-keeper”, limiting the PAS domain cavity access and EAG1 current inhibition by imipramine. In contrast, the hydrophobic profile of the cavity, and residues D39 and R84 flanking the entrance to the cavity facilitated EAG1 current inhibition by imipramine. These results provide insight into the molecular mechanism of EAG1 channel inhibition by imipramine binding to the PAS domain.

While it has been well established that proteins containing the PAS domain structural fold serve as ligand-binding modules, coupling the protein function to changes in the ligand concentration during cellular signaling (28, 29, 36), the ligand-binding capacity of the PAS domains of EAG and related channels was revealed only in the last 5 years. Undecylenic acid, chlorpromazine, and now imipramine, have been shown to directly bind to the PAS domains of EAG channels and inhibit EAG currents by our research group (24, 25). In addition, heme was found to directly bind to the PAS domain of ERG3 channels and inhibit these channels by another research group (26). These recent studies indicate that there is an untapped potential for ligand regulation of KCNH channels *via* small molecule binding to their PAS domains. This regulation can be exploited for therapeutic purposes and it is still possible that some naturally occurring signaling molecules could regulate the activity of these channels *via* binding to their PAS domains in the native cellular environment.

Before this study, the functional link between the direct binding of small molecule ligands to the PAS domain and current inhibition was only supported by the decrease in current inhibition by chlorpromazine for EAG1 channels lacking the PAS domain (25). However, a truncation of the entire domain can have substantial allosteric effects on the channel gating that could mask the molecular mechanisms of the small molecules' action. Here we used MD simulations to guide our mutagenesis and electrophysiology experiments to identify specific residues on the PAS domain that contribute to the effect of the PAS domain small molecule binders on EAG1 currents. Using this approach, we identified residues on the PAS domain that hinder (Y71) and facilitate (D39 and R84) EAG1 current inhibition by imipramine. Importantly, the substitutions of these residues with glycine had little effect on the current profile and voltage-dependence of EAG1 channel activation (Fig. 5), suggesting that the general channel gating mechanism was not affected by the point mutations. Our MD simulations and computational analysis indicated that imipramine binding is driven by hydrophobic interactions between the hydrophobic residues in the cavity and the hydrophobic rings of imipramine.

In the PAS domain structure, Y71 is partially occluding the entrance to the PAS domain cavity limiting access of ligands such as imipramine to the cavity (Fig. 2*D*). The MD simulations show that in order to accommodate imipramine, Y71 has to move away from the cavity. This suggests that Y71 functions as a gate-keeper residue limiting access of imipramine to the cavity. In agreement with this, the substitution of Y71 with structurally similar phenylalanine did not affect EAG1 current inhibition by imipramine, while the substitution of Y71 with a smaller glycine or valine increased EAG1 current inhibition by imipramine. Since chlorpromazine is structurally similar to imipramine, most likely its binding to the PAS domain inhibits EAG1 currents *via* a similar mechanism. Interestingly, a tyrosine residue of Aryl hydrocarbon Receptor (AHR) transcription factor PAS domains, located at the position equivalent to the one of Y71 in the PAS domain of EAG1 channels, was also proposed to function as a “gatekeeper” residue regulating access and affinity of the ligands (37), suggesting that this function of the tyrosine residue is conserved among the PAS domains even in proteins with seemingly different functions.

**Figure 6:**
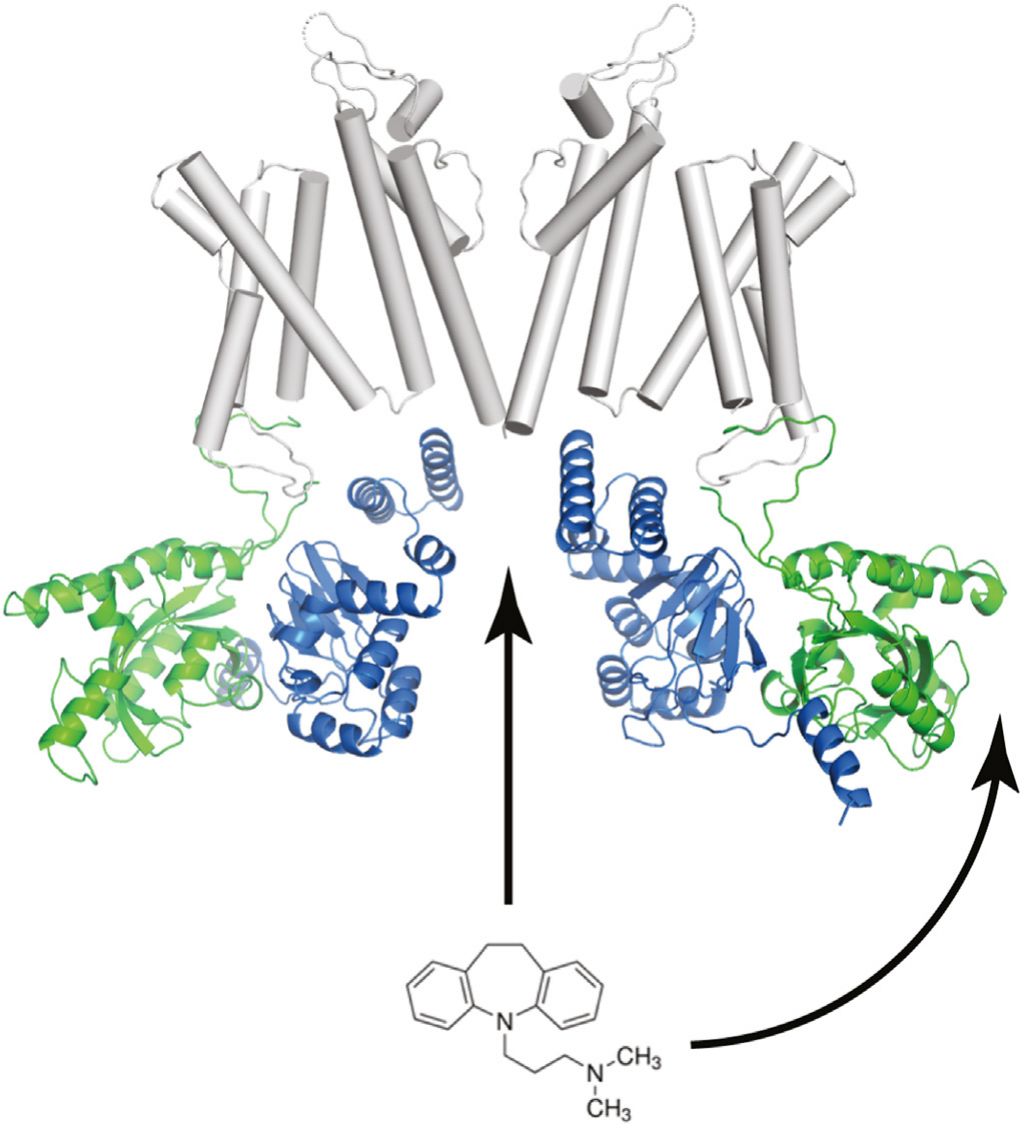
**A model of EAG current inhibition by imipramine by a dual mechanism, *via* binding to the PAS domain and the intracellular pore vestibule.***Ribbon* representation of the full-length cryo-EM structure of rat EAG channels (PDB 5K7L) viewed from the side. Only two diagonal subunits are shown for clarity. The PAS domains are *green*, the CNBH *blue*, and the transmembrane segments *gray*. The *arrows* signify imipramine binding to the PAS domain and intracellular pore vestibule.

The deletion of the PAS domain and D39G/R84G double mutation decreased EAG1 channel inhibition by imipramine equally (Table 1), indicating that D39 and R84G are essential for EAG1 current inhibition by imipramine binding to the PAS domain. Importantly, for both the PAS domain deletion and D39G/R84G mutation, EAG1 currents were still inhibited by imipramine, although with a two-fold higher IC_50_ (Table 1). It has been shown that imipramine also inhibits EAG1 channels in a voltage-dependent manner and competes for this inhibition with an open-pore channel blocker TEA (27). Taken together with the findings of our study, this suggests that imipramine can inhibit EAG1 channels *via* a dual mechanism: by binding to the PAS domain and by entering the intracellular vestibule and blocking currents through EAG1 channels (Fig. 6). We hope that the molecular mechanism of imipramine inhibition uncovered in our study will facilitate the development of EAG1 channel-specific small molecule inhibitors that bind to their intracellular PAS domains and can be used for the treatment of cancer and neurological disorders associated with defects in EAG1 channel function.

The IC_50_ value for imipramine inhibition of WT EAG1 channels determined with TEVC (∼60 μM) was higher than the IC_50_ determined for EAG1 currents recorded from HEK293 in the whole-cell configuration (∼2 μM) (27). This is, at least in part, due to the much larger size of oocytes compared to mammalian cell lines. The large size of oocytes leads to an excessive buffering of applied ligands and decreases the effective ligand concentration. Imipramine is approved by the Federal Drug Administration for use as an antidepressant. The plasma levels of imipramine observed in patients can reach up to ∼300 ng/ml (∼1 μM) (38, 39). Moreover, due to the high membrane permeability, tissue accumulation, and prolonged use, the effective cellular concentration of imipramine is expected to be 10 to 1000-fold higher (40, 41, 42, 43). Therefore, the IC_50_ of EAG1 channel inhibition should be within the clinically relevant doses of imipramine. Since EAG channels are abundantly expressed in the brain (44, 45, 46), their inhibition by imipramine at the clinically relevant doses should be highly physiologically relevant.

Gain-of-function mutations in EAG1 channels are associated with severe neurological disorders, including epilepsy, Zimmerman–Laband and Temple–Baraitser syndromes (5, 6, 7, 8). Moreover, upregulation of EAG1 channel activity is associated with cancer (10, 11, 16, 47, 48, 49, 50). Therefore, as FDA-approved medications, imipramine, and chlorpromazine could potentially be repurposed for the treatment of neurological disorders and cancer. In addition, due to the abundant expression of EAG1 channels in the brain, inhibition of their activity by imipramine and chlorpromazine could be underpinning some of the off-target effects observed in patients and also contribute to the principal effect of these therapeutics.

## Experimental procedures

### Electrophysiology

The cDNA encoding wild-type (WT) mouse EAG1 channels in pGH19 vector was kindly provided by G. Robertson (University of Wisconsin-Madison, Madison, WI). The mutant ΔPAS EAG1 channel in pGH19 with the deletion of the PAS domain residues 2 to 130 was generated by Bio Basic Inc and verified by DNA sequencing (Genewiz). Mutant EAG1 channels with single amino acid substitutions were generated by Genewiz and verified by DNA sequencing. cRNA was transcribed using the T7 mMessage mMachine kit (Thermo Fisher Scientific). Defolliculated *Xenopus laevis* oocytes were purchased from Ecocyte Bioscience or *Xenopus*1 and injected with the cRNA using a Nanoinject II oocyte injector (Drummond).

For EAG1 current recordings, oocytes were placed into a handmade chamber containing a bath solution for the current recording. The currents were recorded using a TEVC technique with OC-725C amplifier (Warner Instruments) and pClamp11 software (Molecular Devices). The signals were digitized using Digidata 1550 (Molecular Devices). Patch pipettes were pulled from borosilicate glass and had resistances of 0.7 to 1.5 MΩ. The recording (bath) solution contained 96 mM NaCl, 4 mM KCl, 0.1 mM CaCl2, 1.8 mM MgCl2, and 5 mM HEPES, pH 7.5. The pipette solution contained 3 M KCl. The WT and mutant EAG1 currents were elicited by applying a series of 0.1-s voltage pulses ranging from −100 to +50 mV in 10-mV increments from a holding potential of −80 mV, followed by a 0.15-s voltage pulse to −100 mV. The currents were not leak-subtracted. Capacitive transients were excluded from the figures for clarity.

Imipramine was purchased from Sigma. Imipramine stocks were prepared in high-purity water and then diluted with the bath solution to obtain the range of concentrations used for the dose-response experiments. The bath solution was changed using a gravity-fed solution changer. To determine the IC_50_, the concentration of imipramine at half-maximal current inhibition, the plots of the percent current inhibition *versus* the concentration of imipramine were fitted with a Hill equation:

(1) $$Y\left[x\right]=Y_{0}+\left(\frac{\left(Y_{\infty }+Y_{0}\right)}{\left(1+{\left(\frac{IC50}{x}\right)}^{n}\right)}\right)$$

where Y_0_ represents the minimum % inhibition, Y_∞_ the maximum, and n is the Hill coefficient.

To analyze voltage-dependence of the steady-state currents recorded from WT and mutant EAG1 channels with TEVC, the conductance (G) was calculated as G = I_ss_/(V −V_rev_), where I_ss_ is the steady-state current recorded at the end of the 0.1 s voltage pulses, V is the test voltage and V_rev_ is the membrane reversal potential for K^+^ selective channels. For our experiments, V_rev_ was −83.9 mV, calculated based on the bath concentration of K^+^ of 4 mM and the intracellular K^+^ concentration of 109.5 mM (51). The conductance was normalized to the largest conductance for the given oocyte (G_max_) in the absence of imipramine. The normalized conductance was then plotted against the test voltage, and the plots were fit with a Boltzmann equation:

(2) $$\frac{G}{G_{\max}}=\frac{1}{1+e^{\frac{V-V_{1/2}}{s}}}$$

where V represents the test voltage (mV), V_1/2_ is the half-maximal activation voltage (mV), and *s* is the slope of the relation (mV).

For each mutant and imipramine concentration, more than five recordings were obtained, each from a different oocyte harvested from at least three different frogs. The ligand concentrations were changed from low to high over the course of the experiments. The absence of detectable currents for F87G/F130G, V80G/F87G, and F87G/F130G double mutant channels was confirmed by recordings from more than 30 different oocytes harvested from at least three different frogs. The error bars on the figures correspond to the Standard Deviation (SD). Statistical analysis was performed using Student’s t-tests. *p* values <0.05 were considered significant. The data analysis and fitting of the plots were performed in Clampfit (Molecular Devices) and Origin (Microcal Software, Inc).

### Replica exchange solute tempering and molecular dynamics simulations

The structural model for the replica exchange solute tempering (REST2) (32) was prepared using CHARMM-GUI (52) using the PAS domain structure (pdb id: 4hoi) (53). Na^+^ and Cl^−^ ions were added to the system at 150 mM concentration. Molecular dynamics (MD) simulations were performed using CHARMM36 (54) forcefield for the protein, TIP3P model for the waters (55) and NAMD program with REST2 support (56). A force-switching function was used for van der Waals and electrostatic interactions between 10 and 12 Å (57) and long-range interactions were computed with the Particle Mesh Ewald (PME) method (58). Langevin piston was used to maintain pressure at 1 bar (59). A timestep of 2 fs was used for the equilibration. An integration time step of 2 fs was used for all simulations using SHAKE algorithm to constrain hydrogen atoms (60). We first ran a 10 ns standard MD simulation to equilibrate the PAS domain. REST simulations ran with 20 replicas between effective temperatures of 310 and 610 K. Replica exchanges were attempted every 2 ps between neighboring replicas along the temperature scale. Each replica ran for 50 ns and the total accumulated simulations time was 1 μs. The protein was chosen as the hot region in REST2 simulations.

To simulate the ligand-bound PAS domain, we selected a snapshot of a replica exchange simulation where the binding pocket in the PAS domain was readily open and the Y71 was no longer blocking the entrance to the PAS domain cavity. Next, we used Autodock Vina (31) to dock imipramine to the binding pocket. This led to the binding of imipramine inside the PAS domain cavity. Follow-up MD simulations were performed using GROMACS 2018 software (61), AMBER99SB-ILDN forcefield for the protein (54), TIP3P model for waters, and parametrization of the ligands with general amber forcefield (GAFF) (62). Na^+^ and Cl^−^ were added to the system to a final concentration of 150 mM. Simulations were performed with a 2 fs timestep at 310K temperature and pressure of 1 bar. A velocity-rescaling thermostat was used to maintain the temperature at 310K. During equilibration, the pressure was maintained at 2 bar using Berendsen barostat. During the production run, the system was simulated under an NPT ensemble with Parinello-Rahman barostat to maintain pressure at 1 bar using a compressibility of 4.5 × 10^−5^ bar^−1^ and a coupling constant of 0.5 ps. The simulations lasted for 100 ns for each complex. Here we used 80 and 2 as the solute and solvent dielectric constants.

### Protein expression and purification

DNA encoding PAS (residues 7–136) of mouse EAG1 channels (GI # Q60603) was synthesized by BioBasic and subcloned into pETM11 bacterial expression vector containing an N-terminal 6-His affinity tag followed by a tobacco etch virus (TEV) protease cleavage site. The DNA sequences were verified by sequencing (Genewiz). The PAS domains were expressed in BL21 (DE3) *Escherichia coli* cells as previously described (25, 63). The cells were grown at 37 °C to an optical density at 600 nm of 0.6 to 0.8, induced with IPTG at 18 °C overnight and harvested by centrifugation. The cells were resuspended in 150 mM KCl, 1 mM TCEP, 1 mM ABSF, 2.5 mg/ml DNaseI and 30 mM HEPES, pH 7.5. Cells were lysed with an Emulsiflex-C5 (Avestin). Insoluble protein was separated by centrifugation in 45 Ti rotor at 30,000 rpm for 1 h at 4 °C. The PAS domains were purified by Ni^2+^ affinity chromatography using HisTrap HP column (Cytiva) and eluted on a linear gradient to 500 mM imidazole. The protein was further purified on a Superdex 200 Increase 10/300 column (Cytiva) equilibrated with 150 mM KCl, 1 mM TCEP, 10% glycerol, and 30 mM HEPES, pH 7.5. The protein concentration was determined with Bradford Protein Assay Kit (Pierce).

The purified protein was stored at −80 °C in aliquots and thawed immediately before the experiments. The molecular weight of the PAS domains used in the study was verified on Coomassie Blue-stained gels and with mass spectrometry at the Proteomics and Metabolomics Core Facility at Georgetown University Medical Center.

### Surface plasmon resonance measurements

All SPR binding experiments were performed on a CM5 chip (Cytiva) at 25 °C using a Biacore T200 Instrument (GE Healthcare). To probe imipramine binding to the PAS domains, the purified PAS domains were immobilized on the CM5 chip using a standard amine coupling chemistry in the presence of 10 mM sodium acetate buffer at pH 5.5 as the immobilization buffer (buffer used to directly dissolve ligands), as we described before (63). HBS-P buffer (150 mM NaCl, 10 mM HEPES, 0.05% (v/v) surfactant P20, pH 7.4) was used as the immobilization running buffer (buffer that runs in the background during immobilization). Imipramine, dissolved in the kinetics running buffer containing (in mM): 150 mM KCl, 1 TCEP, 10% Glycerol, 30 HEPES, pH 7.5 and supplemented with 0.05% Tween 20, was then injected over the immobilized PAS domains over the range of concentrations in triplicate for 60 s at a flow rate of 30 μl/min, followed by buffer only injections for 150 s. The SPR data were doubly corrected: First, a reference flow cell (FC) was activated and deactivated, using the same standard amine coupling chemistry, as in active FC with immobilized proteins, but without a protein. This reference FC was used as a reference surface to account for a potential non-specific binding to the chip surface; Secondly, binding corresponding to blank injections (kinetics running buffer only) was subtracted from the reference subtracted SPR data. Imipramine displayed non-specific binding to the CM5 chip surface at a concentration ≥100 μM. Therefore, the effect of imipramine was examined at concentrations ≤30 μM. Each experiment was repeated on three different CM5 chips.

## Data availability

All data are contained in the article or available on request by contacting the corresponding authors: tib5@georgetown.edu or jbklauda@umd.edu.

## Supporting information

This article contains supporting information.

## Conflict of interest

The authors declare that they have no conflicts of interest with the contents of this article.
