# Supplementary material for: Molecular mechanism of EAG1 channel inhibition by imipramine binding to the PAS domain
Source: J Biol Chem. 2023 Oct 28;299(12):105391. doi: 10.1016/j.jbc.2023.105391 (PMC10687071; doi:10.1016/j.jbc.2023.105391)
Supplement: Supplementary Fig. 1 [file mmc1.pdf]

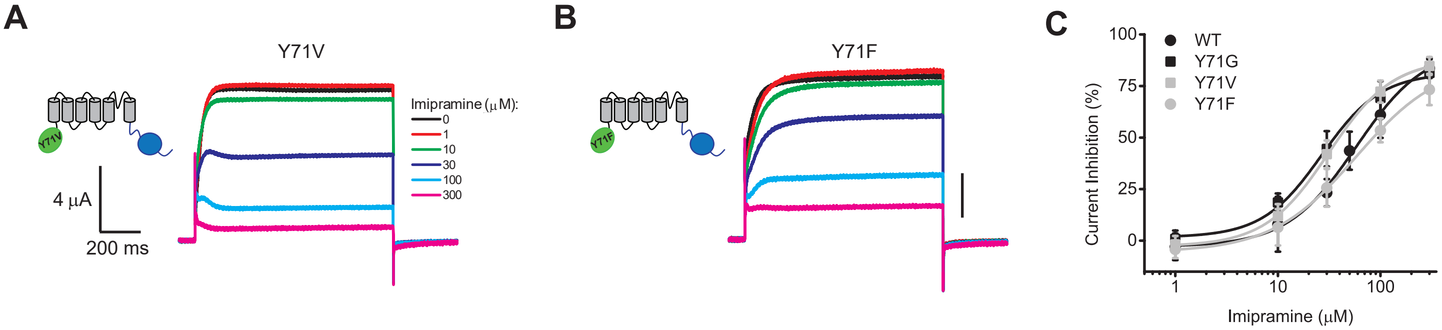

Figure S1. Y71 restricts binding of imipramine to the PAS domain cavity. Currents from Y71V (A) and Y71F (B) mutant EAG1 channels recorded at +50 mV with TEVC in the presence of the indicated imipramine concentrations. C, plots of the averaged percentage of steady-state current inhibition versus imipramine concentration for Y71V (grey squares, n = 6) and Y71F (grey circles, n = 6) mutant EAG1 channels, and WT (black circles) and Y71G (black squares) mutant EAG1 channels from Fig. 2B. The lines correspond to the fits with the Hill equation. The IC50 values and the corresponding statistical analysis can be found in Table 1. Scale bar in B: 4 μA.
